# Correlation of Various Sleep Patterns on Different Types of Memory Retention: A Systematic Review

**DOI:** 10.7759/cureus.42294

**Published:** 2023-07-22

**Authors:** Purva Dahat, Stacy Toriola, Travis Satnarine, Zareen Zohara, Ademiniyi Adelekun, Kofi D Seffah, Lana Dardari, Korlos Salib, Maher Taha, Safeera Khan

**Affiliations:** 1 Medical School, St. Martinus University, Williemstad, CUW; 2 Research, California Institute of Behavioral Neurosciences & Psychology, Fairfield, USA; 3 Pathology, California Institute of Behavioral Neurosciences & Psychology, Fairfield, USA; 4 Pediatrics, California Institute of Behavioral Neurosciences & Psychology, Fairfield, USA; 5 Internal Medicine, California Institute of Behavioral Neurosciences & Psychology, Fairfax, USA; 6 Family Medicine, California Institute of Behavioral Neurosciences & Psychology, Fairfield, USA; 7 Internal Medicine, California Institute of Behavioral Neurosciences & Psychology, Fairfield, USA; 8 Internal Medicine, Piedmont Athens Regional Medical, Athens, USA; 9 General Practice, El Demerdash Hospital, Cairo, EGY; 10 General Practice, California Institute of Behavioral Neurosciences & Psychology, Fairfield, USA

**Keywords:** mental wellbeing, neuronal correlation, sleep problems, cognitive neuroscience, electroencephalograph (eeg), sleep quality, sleep deprivation, sleep physiology, short-term memory, memory retention

## Abstract

Sleep has a substantial impact on memory consolidation, although the link between specific sleep patterns and different forms of memory retention is not well-understood. The purpose of this systematic review is to investigate the correlation between varying sleep habits and memory recall. To identify pertinent research published between 2017 and 2023, a thorough check of electronic databases was carried out. Inclusion criteria encompassed peer-reviewed articles published in English, focusing on human participants, and investigating the relationship between sleep patterns and memory retention. Data extraction and quality assessment were performed on selected studies. This research used different strategies and examined several forms of memory retention, including declarative memory, procedural memory, and emotional memory. Several sleep patterns, including sleep duration, sleep stages, and sleep continuity, were investigated. This comprehensive study demonstrated the relationship between adequate sleep duration and memory consolidation, particularly in regard to declarative memory. Furthermore, deep sleep, characterized by slow-wave sleep (SWS), has been associated with superior procedural memory retention. Sleep continuity, as evaluated by reduced sleep fragmentation or undisturbed sleep, influenced memory consolidation across multiple categories of memory. However, the relationship between rapid eye movement (REM) sleep and memory retention remains inconclusive due to conflicting findings. This systematic review emphasizes the significance of various sleep patterns in memory retention. Memory consolidation corresponds with adequate sleep length, deep sleep (or SWS), and sleep continuity. Future research ought to investigate the connection between REM sleep and memory retention. Understanding the impact of specific sleep patterns on memory processes might help guide therapies and interventions to improve memory consolidation and overall cognitive functioning.

## Introduction and background

“In terms of memory, then, sleep is not like the bank. You cannot accumulate a debt and hope to pay it off at a later point in time. Sleep for memory consolidation is an all-or-nothing event” [1].

People's ability to recall and forget things is known to be influenced by sleep and emotion. According to earlier studies, emotional or high-arousal stimuli are readily compiled during sleep as opposed to an equal amount of wakeful time. This is in contrast to neutral or low-arousal stimuli. However, two latest meta-analyses found that, while sleep does not preferentially consolidate emotional memories above neutral memories, there are substantial moderators (such as cognitive testing, as to if sleep deprivation (SD) or daytime awake as control conditions) that modify such effects [2]. Recent statistics show that 75% of people with depression experience sleeplessness, and 40% of those who experience insomnia are also thought to be afflicted by a mental health condition [3]. Moreover, poor sleep quality, SD, and sleep disorders may affect one's ability to think clearly and pay close attention [4]. However, it has been demonstrated that the acoustic enhancement of slow waves while sleeping enhances people's working memory (WM) [5]. Another study found that short sleep impairs auditory novelty processing, implying that, when performing a WM task, unrelated auditory input may be less distracting than doing so while being sleep-deprived [6].

As per a survey in March 2023, age-adjusted data on SD by race and ethnicity showed considerable variances; 46.3% of Native Hawaiians and Pacific Islanders, 45.8% of Blacks, 40.4% of American Indians and Alaska Natives, 37.5% of Asians, 34.5% of Hispanics, and 33.4% of Whites reported that they obtain less than seven hours of sleep every night. Within the US, these numbers add up to 35.2%, with Camden (New Jersey) and Detroit (Michigan) at a tie for hosting the most number of people (49.8%) who, on average, gain less than seven hours of sleep every night. Boulder (Colorado) showed to be the place with most people practicing seven hours or more sleep per night. In addition, 57.8% of kids in middle school and 72.7% of students in high school sleep less than eight to 10 hours a night, which is the suggested period for their age [3]. Besides putting in the recommended hours, having quality sleep is also equally, if not more, important. This could be assessed by the Pittsburgh Sleep Quality Index (PSQI).

The PSQI is a standardized subjective questionnaire to evaluate the quality of sleep over the span of a month. It consists of seven scales: sleep latency, sleep duration, sleep disturbance, sleep efficiency, overall sleep quality, daytime dysfunction due to sleepiness, and sleep medication use [7]. Each attribute receives a score between zero and three. The PSQI's global score is obtained by adding the scores of the seven components, yielding a general score ranging from 0 to 21, with fewer points indicating better sleep quality. A participant is deemed to have poor sleep quality if their overall PSQI score is greater than five [8].

The majority of experimental studies investigating the effects of sleep loss on mental function have focused on performance following a night of complete SD, which adversely affects a variety of cognitive functions. WM and executive function, which rely on prefrontal cortex function, are particularly susceptible to complete SD. Although the effects of SD on growth and academic performance can be severe, it is still unclear how much SD causes cognitive impairment, which cognitive functions and underlying neurophysiology are most negatively impacted, and how much therapies such as daytime naps can help make up for these deficits [9]. Adolescents who say they nap a minimum of once per week fall into the category of "habitual nappers," which, according to a recent study, indicates to be 40%-60% of them. Teenagers also take more naps during the week than on the weekends. This is consistent with the observation that more frequent daytime naps are linked to shorter nocturnal sleep. Studies show that those who nap regularly may experience greater stage 1 sleep throughout naps and feel more rested when they wake up, whereas people who nap infrequently have high levels of the deep state of the non-rapid eye movement (NREM) sleep phase, N3 [10]. It is believed that short-wave sleep (SWS) and related slow-wave activity (SWA) is crucial for reducing synaptic strengths, allowing overloaded synapses to reset and promoting experience-dependent learning while awake [6].

Given that a person takes approximately 90-110 minutes to go through the four phases of sleep and rapid eye movement (REM) sleep is the last level attained during each cycle, short naps (under 60 minutes) usually prevent people from getting enough REM sleep. Since the studies conducted by far are only done on subjects’ total SD or brief naps, the role of REM sleep on memory consolidation remains yet to be discovered. Moreover, further studies are necessary to account for people who are habitual nappers than those who are not, in studies with brief sleep periods, or people who are chronic insomniacs than those who are not in SD studies.

A good night's sleep is defined as falling asleep easily, not fully waking up during the night, not waking up too early, and feeling refreshed in the morning. It is not usual for healthy adults of any age to have difficulties falling asleep or sleeping through the night on a regular basis. [11]. In this systematic review, we aim to investigate the numerous variables that determine sleep duration and quality, as well as their impact on our cognitive ability.

## Review

Methodology

*Reporting Guidelines* 

To conduct this systemic review, the Preferred Reporting Items for Systemic Review and Meta-Analysis (PRISMA) 2020 standards were followed [12].

Search Sources and Strategy

We began our search between February 12 and February 19, 2023. We searched through PubMed, PubMed Central (PMC), EBSCO, and Google Scholar to find pertinent literature. We used several combinations of memory retention, short-term memory, sleep studies, and sleep patterns to search all databases. However, the following technique was created and applied in PubMed, in addition to these keywords, to search for pertinent literature in the MeSH database: ((( "Sleep/genetics"[Majr] OR "Sleep/physiology"[Majr] )) AND (( "Memory, Short-Term/classification"[Majr] OR "Memory, Short-Term/physiology"[Majr] ))) AND (( "Memory/classification"[Majr] OR "Memory/physiology"[Majr] )). Boolean operators AND, OR, and NOT were used to narrow the results of searches in different database search engines to papers published between 2018 and 2023. The databases that were used and the estimated quantities of papers for each database are displayed in Table 1.

**Table 1:** Keywords/strategy used to screen the databases and the number of papers identified MeSH: Medical subject heading

Keywords and search strategy	Database used	Number of papers identified
Short-term memory AND sleep	PubMed	1,165
((( "Sleep/genetics"[Majr] OR "Sleep/physiology"[Majr] )) AND (( "Memory, Short-Term/classification"[Majr] OR "Memory, Short-Term/physiology"[Majr] ))) AND (( "Memory/classification"[Majr] OR "Memory/physiology"[Majr] ))	PubMed-MeSH	68
Varied sleep duration AND short-term memory retention	PMC	2,131
Sleep breaks AND cognitive memory recall functioning	EBSCO	941
Sleep breaks AND short-term memory retention NOT long	Google Scholar	16,900
Total number of papers identified		21,205
Total number of papers after removing duplicates and narrowing down to review the studies within the last 5 years only		786

Eligibility Criteria

The papers found in the above-mentioned databases were further screened to obtain a final number of studies that were referenced for this systematic review. The eligibility criteria for the same are shown in Table 2.

**Table 2:** Table summarizing the inclusion and exclusion criteria

Inclusion criteria	Exclusion criteria
Articles within the last 5 years	Participants not on caffeine, alcohol, and stimulants for >24 hours or on any medications
Papers published in English	People suffering from intellectual developmental delay & sleep disorders
Papers including only sleep studies or only memory retention or both	Studies on non-human subjects
Randomized control trails	Grey literature
Full-text articles	People with pathologic cognitive function decline
Peer-reviewed articles	Studies on <18 y/o

Screening Process

We identified a total of 21,205 relevant papers with the databases mentioned above. Next, we eliminated any duplicate papers and moved the shortlisted articles to Endnote and Google Sheets. Titles and abstracts were used to examine each publication for eligibility independently. Only pertinent articles were analyzed for the entire text. Only items that met the criteria for inclusion and exclusion were reviewed. The chosen subjects were then evaluated for quality.

Quality Appraisal

Article selection, evaluation, and analysis were conducted by two distinct investigators (the first and second authors) at each stage. The group's consensus was used to reevaluate an article's full text if there were any discrepancies in the results about its eligibility. In case of conflict, a third reviewer was involved. Using the appropriate quality assessment techniques, the shortlisted articles were evaluated. As per the inclusion criteria, all the selected papers were randomized controlled trials (RCTs), and their quality was assessed by using the Cochrane bias assessment tool. In this review of the literature, only studies that met the quality appraisal criteria of 70% or more were considered.

Results 

Study Identification and Selection

**Figure 1:**
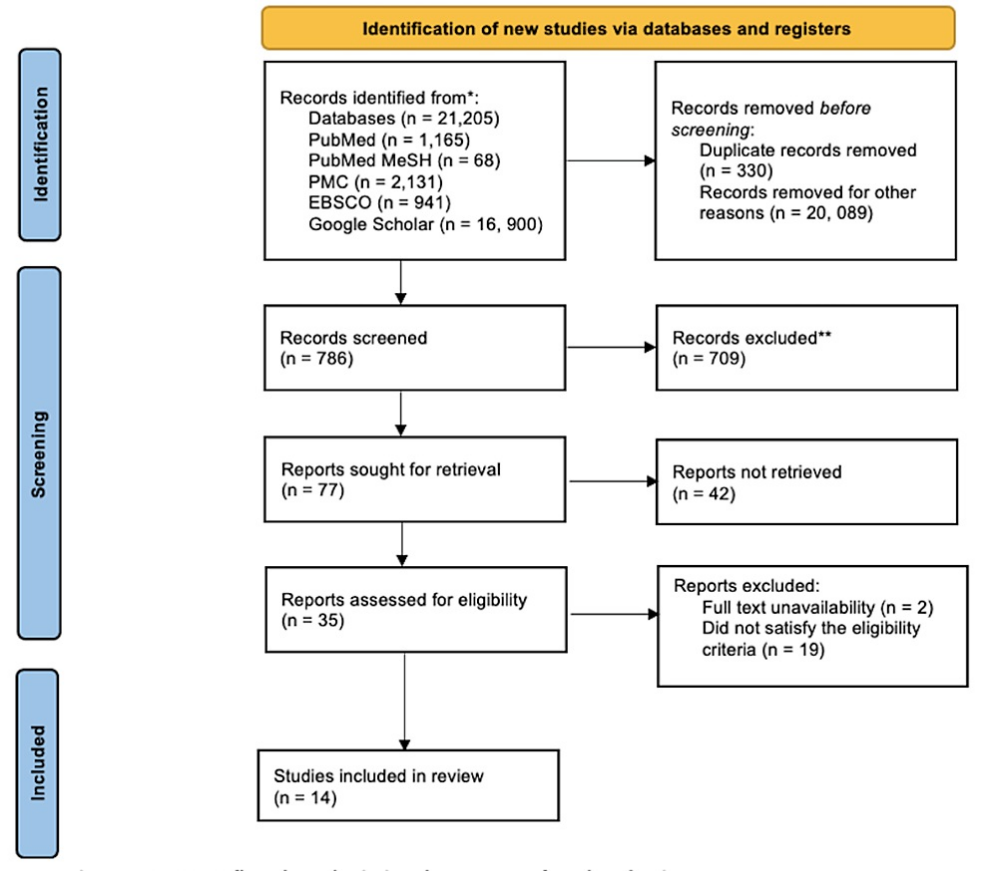
PRISMA flowchart depicting the process of study selection PRISMA = Preferred Reporting Items for Systemic Review and Meta-Analysis

Using all databases, we identified an overall of 21,205 relevant articles. Before thoroughly analyzing them, 330 duplicate articles and 20,089 items dated more than five years were eliminated. Consequently, 35 papers were shortlisted after being screened by looking at the titles and abstracts and getting the full texts, and 14 final full-text papers from the final list were chosen for review after they underwent quality and eligibility checks. Figure 1 displays the PRISMA flowchart that illustrates how the studies were chosen.

All papers were independently assessed to minimize the possibility of bias; we assessed every article's quality by using the Cochrane bias assessment tool for RCTs. We closely reviewed each article for the seven potential sources of bias, namely, random sequence generation, allocation concealment, participant blinding, outcome assessment participant blinding, selective reporting, incomplete outcome data, and other bias. We classified each potential source of bias into three categories: low risk of bias, high risk of bias, and ambiguous risk of bias. Table 3 presents the results of the quality appraisal.

**Table 3:** Quality appraisal using the Cochrane bias assessment tool + : low risk, - : high risk, ? : ambiguous risk, N/A: not assessed

Authors and year of publication	Random sequence generation	Allocation concealment	Blinding of participants and personnel	Blinding of outcome assessment	Incomplete outcome data	Selective reporting	Other bias
Zeng et al. 2021 [2]	+	+	?	+	+	+	N/A
He et al. 2020 [4]	+	-	+	+	+	+	-
Wofford et al. 2022 [5]	+	+	+	+	+	+	+
Rångtell et al. 2019 [6]	+	+	+	+	-	-	+
Cousins et al. 2019 [9]	+	-	+	+	+	+	-
Leong et al. 2021 [10]	+	+	+	+	+	+	?
Snipes et al. 2022 [13]	+	-	+	+	+	+	?
Mograss et al. 2020 [14]	+	+	+	+	+	?	+
Zhu et al. 2019 [15]	+	-	+	+	+	+	N/A
Guo et al. 2019 [16]	+	-	+	+	?	+	+
McMahon et al. 2018 [17]	+	-	?	+	+	+	+
Wang et al. 2021 [18]	+	-	+	+	+	+	-
Chen et al. 2021 [19]	+	+	+	+	+	+	-
Dehnavi et al. 2019 [20]	+	+	-	+	+	+	N/A

All papers that were referenced for this article surpassed the quality assessment by 70% and more.

Outcomes Measured

The primary outcome extracted from the finalized research papers is that sleep plays a crucial role in various cognitive and emotional processes, including memory consolidation, emotion regulation, decision-making, and social interactions. The secondary outcomes investigated included the impact of sleep on neural connections and brain processes, particularly in the prefrontal cortex, hippocampus, and amygdala. Prolonged SD and sleep disorders can impair these cognitive and affective functions and severely impact one's health by increasing the risk of obesity, diabetes, cardiovascular disease, and mental problems. Consequently, encouraging proper sleep hygiene and making sure that one gets enough sleep, both quantity-wise and quality-wise, should be top priorities for preserving one's health and well-being.

Study Characteristics

We reviewed 14 research papers with a total of 646 participants. Although all these finalized studies [2,4-6,9,10,13-20] were RCTs, among them, they include research on the relationship between sleep and cognitive function, mental health, physical health, and sleep disorders. The studies utilize a variety of methodologies, including experimental interventions and neuroimaging techniques. Many of the studies have focused on specific populations, such as adolescents and older adults. Overall, the studies highlight the importance of sleep for physical and mental well-being and suggest that improving sleep quality may positively impact a range of aspects of health and behavior. Table 4 shows the summarization and characterization of all the studies included in this review.

**Table 4:** A summary of all the studies included in this review ERPs: event-related brain potentials; EEG: electroencephalogram; STM: short-term memory; SD: sleep deprivation; NREM: non-rapid eye movement; SWMT: Sternberg working memory task; rMS: recurrent transcranial magnetic stimulation; ALFF: amplitude of low-frequency fluctuations; PVT: psychomotor vigilance test; WMZ: wake maintenance zone; MF: motivated forgetting; S2: stage 2; SWS: slow wave sleep

Authors and year of publication	Purpose of the study	Number of participants	Result	Summary of findings
Zeng et al. 2021 [2]	To study the effects of sleep on preserving short-term memory and enhancing the long-term affect in the consolidation of emotional memories	59	In a 12-hour post-encoding test, sleep impartially retained both neutral and negative memory, as well as affective tones, compared to sleep deprivation. Neutral and negative memories dramatically decreased in the sleep group over the 60-hour post-encoding test, and emotional reactions to unfavorable memories diminished with time. Multivariate whole-brain event-related brain potentials (ERPs) analysis further revealed that sleep prioritized neuronal characterization of emotion above memory processing. However, they were harder to discriminate in the group that had been sleep-deprived.	These findings revealed that sleep had a time-dependent effect on emotional memory and affective responses: short-term memory retention and affective tones were improved by sleep, whereas long-term affective tones were improved.
He et al. 2020 [4]	To analyze the impact of curtailing mobile phone use before sleep on working memory, arousal, and mood	38	The four-week ban on using mobile devices before bed improved working memory and positive affect as well as decreased pre-sleep arousal and increased sleep length, quality, and quantity.	Limiting mobile phone use before bed decreased pre-sleep agitation and sleep delay, increased sleep duration, and improved working memory. Individuals with sleeping problems were advised to make this easy shift to moderate consumption.
Wofford et al. 2022 [5]	To determine whether an acute stressor's detrimental influence is improved by a quick nap	70	Only the sleep group demonstrated a decrease in negative affect when compared to initial negative affect levels. The nap group showed lower negative affect following the break than the waking group.	The results imply that short naps may be a helpful strategy to enhance the mood while dealing with an acute stressor since they show that napping can lessen unpleasant emotions that accompany a stressor rather than taking a break.
Rångtell et al. 2019 [6]	To see if objective working memory performance is sex-dependently impaired by a single night of sleep loss, but not subjective working memory	24	We discovered that sleep deprivation reduced women's working memory performance objectively but not subjectively. In contrast, sleep loss had no impact on either measurement in men. Working memory performance was hampered by auditory distraction without being affected by lack of sleep or sex.	As demonstrated in the study, being ignorant of cognitive limits when sleep-deprived may have negative effects, such as in the workplace. Our findings imply that young women who lack sleep are particularly susceptible to overestimating the effectiveness of their working memory.
Cousins et al. 2019 [9]	To study if short-term topographical memory is saved by a split sleep schedule following several nights of sleep deprivation	118	In comparison to the 9.0 hours control group, the performance of the night-time time-in-bed groups performed at 5.0 hours and 6.5 hours was dramatically reduced. Participants' performance on the split-sleep regimen (5.0 hours + 1.5 hours) was comparable to controls.	The study implies that mild multi-night sleep restriction has an effect on hippocampus function, but that deficits can be reduced by dividing sleep, at least for a short time after rising from a daytime nap. While this divided sleep schedule should not be used in place of getting enough nocturnal sleep, it does seem to help those who are constantly sleep-deprived with their cognitive and neurophysiological processes that underlie learning.
Leong et al. 2021 [10]	To understand memory function after a nap in regular and sporadic nappers	92	Whether or not one regularly naps, an afternoon nap improved visual encoding and factual knowledge learning. On the hippocampal-dependent topographical memory task, however, we discovered a significant interaction wherein sleep, relative to wake, benefited habitual nappers over non-habitual nappers. Significantly, when given the chance to nap regularly, habitual nappers' performance drastically decreased.	We discovered that an afternoon sleep was beneficial for long-term memory tasks even if one did not regularly nap. When habitual nappers were asked to do a task requiring short-term topographical memory, naps were especially helpful because they prevented the deterioration that would otherwise have occurred. The use of naps in educational settings to improve and safeguard adolescent students' learning and memory is supported by research.
Snipes et al. 2022 [13]	To explore the theta paradox: EEG oscillations between 4 and 8 Hz reflect both sleep pressure and cognitive control	18	Theta power was shown to be enhanced by both cognitive load and sleep deprivation in medial prefrontal cortical regions, while sleep deprivation also led to additional increases in theta in numerous other areas, primarily frontal ones. A visual-spatial task and a short-term memory (STM) task showed the most noticeable effects of sleep deprivation (SD) theta, which was task-dependent. Importantly, theta was highest during a spatial game and highest in the inferior temporal cortex (associated with object recognition) during passive music listening.	Theta oscillations throughout cognition and theta throughout sleep deprivation did not differ significantly in our research, ruling out the possibility that they serve separate purposes. Our findings show that theta oscillations in both instances are instead produced by cortical regions that are not necessary for continued behavior. Theta may therefore represent cortical inhibition or disengagement, at least in humans.
Mograss et al. 2020 [14]	To see if memory is improved by exercising before napping more than by napping or exercising alone	115	In the visual recognition test, participants who had both exercised and a nap performed much better than those who had either napped or exercised. Higher identification accuracies were connected to increased sleep spindle densities in the combined nap and exercise group.	Recognition memory is improved more by combining a Non REM nap and a short bout of intense aerobic exercise than by either napping or exercising alone. Sleep and exercise together improve long-term memory rather than being distinct elements that affect it separately.
Zhu et al. 2019 [15]	To explore neural correlates of dynamic variations in the efficiency of working memory after one night without sleep	36	We discovered that the weakest brain responses and the slowest Sternberg working memory task (SWMT) reaction times occurred early in the whole sleep deprivation period rather than at the end of the total SD. Moreover, during this most severe total SD stage, reaction times for SWMT were found to be positively correlated with the magnitude of negative correlation between the control and default networks and negatively correlated with task-related activation in the angular gyrus.	Further evidence that different cognitive activities are affected differently by sleep deprivation and circadian rhythmicity was revealed by the rebound of Sternberg’s working memory task (SWMT) reaction time and cerebral responses just after the mid-time point of the usual biological sleep night.
Guo et al. 2019 [16]	To analyze whether poor working memory caused by sleep deprivation may be improved by high-frequency repetitive TMS	17	When given working memory tests involving letters and numbers after sleep deprivation, the individuals' response accuracy was worse and their reaction times were longer. Following recurrent transcranial magnetic stimulation (rTMS), the response accuracy of numbers considerably increased, mirroring the refreshed waking state following a typical night of sleep. The frontal gyrus, precuneus, angular gyrus, and parietal lobe showed significantly enhanced amplitude of low-frequency fluctuations (ALFF) following rTMS, although ALFF reduced from the relaxed waking period state to the condition of sleep deprivation in these brain regions.	Findings suggest that, by altering the neuronal activity of associated brain areas, high-frequency recurrent transcranial magnetic stimulation delivered over the left dorsolateral prefrontal cortex may aid in the recovery of poor working memory following sleep deprivation.
McMahon et al. 2018 [17]	To investigate how the effect of sleep deprivation on cognition is modulated by the relationship between homeostatic sleep pressure and circadian timing impacts various cognitive processes	23	Except for recognition memory, performance on most measures is impacted by prolonged awakeness. Performance on all metrics was much worse after 27 hours of sleep deprivation than it was 24 hours earlier when well-rested. Although vigilant attention and psychomotor vigilance test (PVT) performance were hampered after 37 hours of awake time, complex attention and working memory were intact. Efficiency and precision were both affected by sleep deprivation in the morning, whereas only speed was affected in the late afternoon and evening.	At 27 and 37 hours of awake time, we saw task- and time-dependent consequences of lack of sleep, which significantly reduced alert attention (in comparison to when well-rested at a similar circadian clock time). Nevertheless, despite higher homeostatic sleep pressure in the wake maintenance zone (WMZ), working memory and complex attention were sustained at 27 hours of awake time (37 hours awake).
Wang et al. 2021 [18]	To examine the impact of sleep, peaceful rest, and awake activity on procedural and declarative memory consolidation	94	For the procedural and declarative memory tasks, sleep and silent rest both produced superior results than the distractor task. Furthermore, the benefits of rest on performance were identical to those of sleep.	These findings show that, at least for extremely brief retention intervals, sleep-specific neurobiology may not be required to trigger memory consolidation. Conversely, given that there is a suitable neurobiological environment and a sufficient reduction in new encoding, offline memory consolidation may operate opportunistically, taking place either during sleep or stimulus-free rest.
Chen et al. 2021 [19]	To demonstrate that working memory and long-term memory are supported by separate offline brain systems that compete with one another	72	We discovered that central activity had a stronger causal effect than autonomic activity. Further, the strength of this impact during sleep led to a behavioral trade-off between offline working memory and long-term memory processing.	The results support the idea that competitive neuronal dynamics occur during NREM sleep and contribute to cognitive improvement. These findings also suggest a system during sleep that switches between the processing of working memory by the vagus nerve and long-term memory by the central brain.
Dehnavi et al. 2019 [20]	To see if motivated forgetting has the opposite effect on sleep spindles in stage 2 and slow-wave sleep	37	We discovered that, compared to the control condition, the no-nap and nap groups remembered considerably fewer no-think terms in the motivated forgetting (MF) condition. For the nap group, spindle strength and density increased throughout stage 2 (S2), whereas it dropped in slow-wave sleep (SWS) in the MF compared with the control condition SWS. Intriguingly, the recall performance of no-think phrases linked positively with spindle power during SWS, but negatively with spindle power during S2.	The startling discovery was that, when compared to spindle activity during SWS, the retention of words that would otherwise be forgotten was inversely associated. These findings show that sleep spindles have a different function in neurocognitive functioning during S2 and SWS and that sleep spindles are responsive to prior MF experiences.

Discussion

The physiological process of sleep is vital to our general health and well-being. It is a critical method that allows the brain and body to unwind and recuperate, ensuring peak mental and physical function the next day. The brain goes through a protracted cycle of physiological changes during sleep that is essential for learning and memory encoding. Even while sleep plays a vital role in our everyday lives, the quantity and quality of sleep we get can be affected by various circumstances. This session will go over recent findings that have improved our understanding of various sleep stages as well as how these sleep stages and sleep disturbances can affect mental performance, psychological health, and overall physical health. We also investigate the physiological and neurological processes that contribute to memory consolidation.

Mechanisms of Sleep and Memory Consolidation in the Neural and Physiological Systems 

Scientific research has extensively examined the interactions between sleep and memory retention. Researchers have made significant progress in comprehending the brain networks and pathways underlying sleep-dependent memory consolidation in recent years, owing to improvements in neuroimaging techniques and algorithmic methodologies [9]. 

The process of consolidating and enhancing new memories in the brain depends heavily on sleep. Several neurological and physiological processes, including neuroplasticity, synaptic potentiation, and the conversion of information from transient to long-term memory (LTM) storage, are thought to play a role in the consolidation of memories while we sleep. Neuroplasticity is the brain's potential to modify and adapt in response to new experiences, and it is one of the basic neuronal mechanisms involved in sleep and memory consolidation. This process, which results in the potentiation of brain connections and the consolidation of new memories, is assumed to happen due to the formation of new synapses and the strengthening of existing ones. Transferring data from transient to permanent memory storage is a crucial physiological process involved in sleep and memory consolidation. Information is primarily encoded in transitory memory storage during awake times, which is prone to deterioration and interference. To avoid loss or degradation over time, this information is instead moved to more reliable and long-term storage during sleep [2].

Memory consolidation during sleep is thought to occur in the hippocampus and neocortex. While the neocortex is essential in consolidating general semantic memories, the hippocampus is crucial in consolidating recent, episodic memories [8]. One study examined the hippocampus and neocortex's effects on memory consolidation during various sleep stages using functional magnetic resonance imaging (fMRI) scans. They discovered that the hippocampus has higher activity during SWS, facilitating information flow from the hippocampus to the neocortex. The neocortex is more active during REM sleep, improving semantic memory consolidation [2]. 

It has also been demonstrated that neurotransmitters, such as acetylcholine and norepinephrine, are crucial for memory consolidation while we sleep. In particular, it has been discovered that the amount of acetylcholine in the brain influences memory consolidation by altering the equilibrium between REM and SWS. Whereas norepinephrine promotes SWS and suppresses REM sleep, acetylcholine stimulates the start of REM sleep. Increasing acetylcholine levels during REM sleep greatly enhanced the consolidation of spatial memory. Another finding was that giving healthy volunteers a norepinephrine reuptake inhibitor increased the retention of declarative memory the following day [20].

Sleep Architecture and Cognitive Correlation

NREM, which can be further subdivided into stage 1 sleep (N1), stage 2 sleep (N2), and SWS (N3), along with REM sleep, constitutes the four stages of sleep. To reset overloaded synapses and enable experience-dependent plasticity while awake, it is assumed that SWS and accompanying SWA are crucial for downscaling synaptic strengths. Recent research indicates that SWS might potentially promote the best cognitive functioning. However, REM might also help lessen stress reactions. During SWS, the hypothalamic-pituitary-adrenal (HPA) axis is inactive; however, during REM, this inhibition is released, leading to comparatively higher blood cortisol. Moreover, during nighttime REM, the levels of both noradrenaline and adrenaline are lowered. For readjusting receptivity to upcoming stressful events and lowering the emotional tone connected to previous stressful events, this decrease may be therapeutic [5].

**Figure 2:**
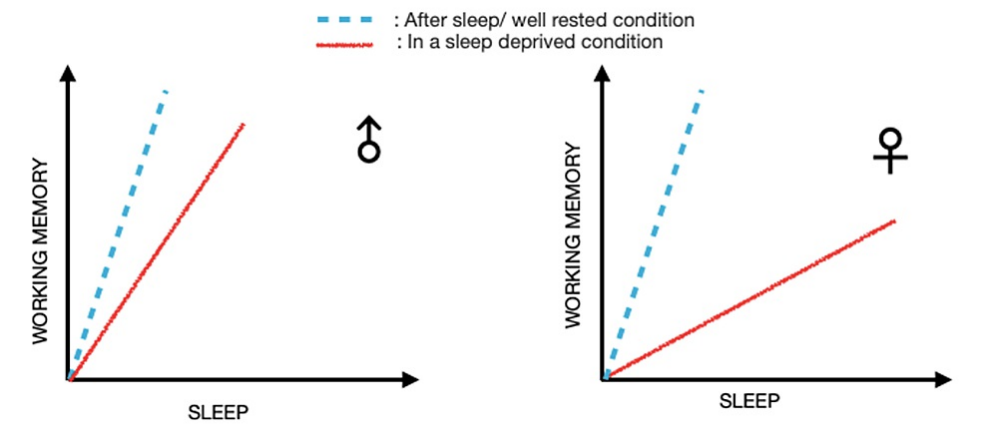
Sleep effect on working memory in a sex-dependent manner

One RCT found that females reported greater perceived stress than males after a stressful event and during a preparation period. This supports previous research showing that women may have stronger negative emotional responses to social stressors than men. However, following a break, stress levels reverted to baseline levels, suggesting that the break was more successful in lowering stress levels in women than in men [5]. Another study also revealed that men and women performed differently regarding WM performance. Women might not be as conscious of how their WM functions after a lack of sleep, which could have major negative effects in real-world situations. Thus, improved sleep may be particularly beneficial for women experiencing disrupted sleep and high WM load [6]. Figure 2 illustrates these findings.

A lack of adequate, high-quality sleep negatively impacts immune function (reduces cytokine production causing increased susceptibility to viruses), psychological issues, increased anxiety and depressive symptoms, increased emotional reactivity and negative affect, exaggerated reactions to stressors (heightened cortisol reactivity), and physical health issues [5]. Mograss et al. further explored this association of sleep and physical health with WM via an experiment wherein he tested on 115 young, healthy participants and realized that higher recognition accuracies in the combined nap with exercise group were correlated with higher densities of sleep spindle. The study concluded that short-term exercise and a nap enhance recognition memory more than a nap or exercise alone [14].

Another study with 17 right-handed undergraduate students in China revealed that, after SD, the subjects' WM assessments for letters and numbers revealed decreased response accuracy and longer reaction times. With recurrent transcranial magnetic stimulation (rTMS), the reduced response accuracy of numbers greatly improved, mirroring the refreshed waking condition following a typical night of sleep. In the brain areas involving the frontal gyrus, precuneus, angular gyrus, and parietal lobe that showed significantly increased amplitude of low-frequency fluctuations (ALFF) following rTMS, and ALFF values fell from the rested waking period state to the state of SD. Moreover, increases in response accuracy and changes in the ALFF values of the inferior frontal gyrus and supramarginal gyrus were found to be significantly positively correlated. These findings suggest that, by altering the neuronal activity of associated brain areas, high-frequency rTMS applied over the left dorsolateral prefrontal cortex (DLPFC) may aid in restoring impaired WM following SD [16]. 

Zhu et al. reported that the anterior cingulate cortex (ACC) is another brain structure that plays a major role in decision-making, attention, and emotion regulation. Over a series of cognitive control tasks, they noted that not only is ACC connected to the insula and the inferior parietal lobule but also that these connections were dynamic. In the sense that depending on the task’s demands, they varied in strength and orientation over time. Furthermore, this strength was linked with variations in cognitive and emotional functioning among people. Particularly, people with a stronger connection between the ACC and the insula exhibited higher levels of stress and anxiety [15]. 

Despite stage 2 (S2) and SWS of NREM sleep, which are two distinct brain states, both contain sleep spindles. In addition to memory retention, it is crucial to comprehend how sleep and eliminating unpleasant memories are related. Dehnavi et al. studied motivated forgetting-dependent alterations in spindle activity separately during S2 and SWS. The startling discovery was that, compared to spindle activity during SWS, the retention of words that would otherwise be forgotten was inversely associated. Fast spindle activity was positively connected with the retention of no-think words during SWS, despite a negative association between no-think word recall performance and spindle activity during S2. These results imply that, in the processing of unwanted memories, sleep spindles play different roles during S2 and SWS [20].

Sleep Deprivation and Short Nap

Electroencephalogram (EEG) oscillations have been linked to many behavioral states, including alertness, drowsiness, and sleep. As a result, oscillations can frequently be employed as quantitative indicators of alertness. Theta oscillations (4-8 Hz) are an exception, as they have been independently recognized as markers of intense cognition and drowsiness. Theta oscillations intensify when people do not get enough sleep. The relationship between circadian rhythm and time spent awake determines when a person feels the need to sleep, and theta is thought to reflect this interaction [13]. 

Snipes et al., in the Journal of Neuroscience, put forth a study that examined how theta oscillations altered when performing cognitive tasks and when deprived of sleep by high-density EEGs in 18 young healthy participants. The study found that both cognitive load and SD increased theta power in the medial prefrontal cortical areas. According to the study, theta power was shown to be elevated in the medial prefrontal cortical regions by cognitive load and SD. However, other frontal regions also experienced an increase in theta due to sleep loss. Task-dependent factors contributed to SD theta, with visual-spatial and short-term memory tasks having the broadest impacts. When passively listening to music and playing a spatial game, theta was the highest in the inferior temporal cortex and supplementary motor areas. Increases in theta were connected with changes in task performance after SD. However, this association was not unique to the EEG of the same task and did not hold up after multiple comparisons correction. With SD and cognition, theta oscillations may preferentially arise in cortical regions that are not involved in ongoing behavior [13].

Zeng et al. showed that, in a 12-hour post-encoding test, sleep similarly preserved both negative and neutral memory, as well as affective tones, compared to SD. Negative and neutral memories dramatically decreased in the sleep group over the 60-hour post-encoding test, and emotional reactions to unfavorable memories weakened with time. These findings revealed that sleep has a time-dependent effect on emotional memory and affective responses: short-term memory retention and short-term amelioration of affective tones were both influenced by sleep. Different neurocognitive processing of distant, emotional memories was found between the sleep and SD groups in both unilateral and multimodal EEG analyses [2].

Another study with 46 participants who napped for 90 minutes and were then given memory tasks demonstrated that, even if one was not a regular napper, taking a nap in the afternoon was better for LTM than staying awake. Regardless of usual napping, a mid-afternoon nap improved visual encoding and factual knowledge learning. On the hippocampal-dependent topographical recall test, however, a significant interaction was discovered wherein sleep, relative to wake, benefited habitual nappers over non-habitual nappers [10]. However, Wang et al., in a recent study, sought to examine the effects of quiet rest, sleep, and alertness on procedural and declarative memory consolidation. Through a series of experiments on 94 subjects, the study concluded that the benefits of quiet rest on performance were identical to those of sleep. These findings show that, at least for extremely brief retention intervals, sleep-specific neurobiology may not be required to trigger memory consolidation. Conversely, given there is a favorable neurobiological setting and a sufficient reduction in new encoding, offline memory consolidation may operate opportunistically, taking place either during sleep or stimulus-free rest [18].

Even though numerous research suggests that naps are good for cognition, it is still unclear whether the benefits of naps themselves or simply having more nighttime sleep can help cognition. A study by Cousins et al. mentioned that, even when total sleep time is regulated, dividing sleep into a nighttime period and a daytime nap enhances hippocampal-dependent cognitive function under chronic sleep restriction. After three nights of relatively light nocturnal sleep restriction of 6.5 hours, 120 participants in this study proved this hypothesis when their performance was impaired and was on par with a more restrictive sleep regimen (five hours nocturnal). In contrast, performance was comparable to a control group that got the recommended amount of sleep for teenagers when sleep was divided between five hours of nocturnal sleep and a 1.5-hour midday nap. Thus, mild sleep restriction for three nights negatively affects hippocampal-dependent topographical memory. Nonetheless, this deficit can be accounted for by dividing sleep into a nocturnal period and a daytime nap. Hence, in conditions of prolonged SD, a split sleep schedule may enhance cognitive and neurophysiological functions related to learning [9].

Impact of Technology-Based Sleep Interventions

Sleep strongly influences the ability to think and feel well. Sleep disorders, SD, and sleep loss may all have an adverse impact on mental and psychological capabilities. Anger, uncertainty, anxiety, and depression were found to be symptoms of sleep disturbances and mood disorders, respectively [4]. Our WM temporarily stores and manipulates information, supporting cognitive functions such as planning, learning, and goal-directed behavior. Acute sleep loss has been found to have a deleterious impact on WM functions, such as vigilance and decision-making [6].

In an experimental study by He et al., all 38 university students in China maintained an online sleep diary, wherein they each submitted their baseline information prior to the intervention and post-intervention data following a week of testing. The participants in the experimental group were then told to put their phones away for 30 minutes before their typical bedtime. After a four-week intervention, sleep latency was reduced by about 12 minutes, and sleep duration was extended by around 18 minutes in the intervention group. Sleep quality was considerably improved Overall, refraining from using mobile devices before night increased the duration and quality of sleep while lowering pre-sleep arousal and negative affect. Moreover, it enhances WM and good affect, making it a beneficial intervention for people with poor sleep quality who wish to scale back on phone use before bed [4].

Another study by Rångtell et al. included 24 young individuals who took part in two experimental conditions that were spaced around one week apart: regular nocturnal sleep (planned between 22:30 and 06:30 hours) and total sleep loss, in that order. Participants were given a digital WM test with 16 trials in the morning following sleep or nocturnal wakefulness, during which eight-digit sequences were learned and recalled. In random order, eight trials of silence and eight trials of auditory verbal distraction were used to teach digit sequences. The study found that, regardless of the quality of sleep, the study discovered that being distracted from spoken Russian sentences had a negative impact on WM. More research is required to explore how other types of auditory distractors and personality measures may affect WM during sleep loss. It is also conceivable that utilizing familiar language as a diversion could have a stronger effect [6].

Performance of the Circadian System in a Task-Dependent Manner

Research explored how circadian timing and SD influence performance outcomes. It implies that circadian timing can have an impact on performance in both positive and negative ways. Even well-rested individuals may perform inadequately during unfavorable circadian times, and sleep-deprived individuals may perform better during favorable hours. The wake maintenance zone (WMZ), which occurs a few hours before melatonin secretion in the evening, is when the circadian system has the most impact on wakefulness and performance [17].

A total of 23 healthy young adults participated in the study, and they strictly followed an 8:16 sleep: wake schedule for the two weeks leading up to the assessment. Actigraphy and their call-ins confirmed that this schedule had been followed. The study includes administering cognitive task tests in the morning and evening and comparing the results to well-rested performance. Simple sustained attention was consistently worse following SD, whereas complex attention and WM were retained in the evening within the WMZ but degraded in the morning. The findings underscore the task- and time-dependent traits of sleep-deprived cognitive function, as well as the relevance of considering circadian rhythms and specific tasks when analyzing performance outcomes [17]. 

To improve operational risk management, the study emphasizes the significance of identifying people susceptible to cognitive failure and the circumstances under which they are most vulnerable.

Mutually Antagonistic Mechanism of Long-Term Emotional Memory (LTEM) and Short-Term Working Memory (STWM) with Sleep 

Word-paired associative (WPA) and operation-span (OS) tasks were used in this study to examine the impact of a pharmacological strategy on long-term memory (LTM) and WM, as well as the bidirectional interaction between central (reflected in sigma activity) and autonomic (reflected in vagal heart rate variability) activities during overnight sleep. A total of 72 individuals who had no prior history of neurological, psychiatric, or chronic disorders participated in two tests. Participants completed sleep diaries and wore activity monitors to ensure they rested before each session. In experiment one, 25 subjects underwent four sessions with zolpidem or a placebo, eight underwent two visits, and one underwent a single visit. In experiment two, 35 people finished the zolpidem night and 36 people finished the placebo night. Also, 33 individuals conducted three sessions of WPA tasks in both circumstances, whereas 35 participants completed three sessions of OS tasks in both conditions. Using effective connectivity defined as the influence one brain system has over another, the model specifically examined whether central sigma activity might decrease autonomic vagal activity [19]. 

Sigma and vagal activity were found to be antagonistic to one another during sleep, and the degree of this reciprocal opposition predicted a behavioral trade-off between LTM and WM. These results suggest that, through alternating between several offline mechanisms, NREM sleep enhances the WM and LTM (i.e., the prefrontal-autonomic inhibitory processing and the hippocampal-cortical dialogue) [19]. 

To that extent, this study proposed a sleep switch model wherein the brain alternates between the two memory processes as a result of a complex interaction at the synaptic (gamma-aminobutyric acid (GABA) versus norepinephrine (NE) activation), systems (thalamocortical versus frontal midbrain), and mechanistic level (sigma-coupled sleep oscillations (SO) versus uncoupled SO). Sigma-coupled SOs facilitate consolidation during the LTM state, resulting in less autonomic vagal-dependent activity and less WM improvement. More efficiency develops in uncoupled SOs during the WM state, which is accompanied by an increase in autonomic vagal-dependent activity. This results in a decrease in central sigma-dependent activity and decreased LTM accumulation [19]. 

The findings of this research imply a system during sleep that shifts between processing LTM by the central brain and WM by the vagus nerve, supporting the hypothesis that competitive neuronal dynamics emerge during NREM sleep and lead to cognitive improvement.

Limitations

Several limitations arise when studying sleep. Self-reporting bias is one of the most common shortcomings since people might not precisely describe their own sleeping habits. Likewise, studies on sleep are frequently undertaken with a limited sample size, which could result in incorrect findings. Moreover, merely momentarily observing sleep can make it difficult to analyze REM sleep, as it occurs around 90 minutes after falling asleep. The lack of control groups in SD studies is another challenge that renders it difficult to compare data accurately. Further, it might be hard to accurately assess sleep duration and quality, which places additional constraints on our ability to comprehend sleep patterns. Last but not least, there are restrictions on the validity and reliability of sleep assessment tools, which may bias outcomes. Overall, these limitations serve as a reminder to researchers to approach sleep research cautiously and to take into account any potential bias.

## Conclusions

This systematic review offers insight into the relationship between different types of sleep patterns and memory retention. The findings suggest that optimal sleep length, defined as a sufficient amount of time, has a beneficial impact on memory consolidation, particularly for declarative memory. Deep sleep, as reflected by SWS, corresponds to better procedural memory retention. Furthermore, sleep continuity, as measured by reduced sleep fragmentation or undisturbed sleep, has a favorable impact on memory consolidation across multiple types of memory. Understanding the importance of specific sleep patterns in memory functions has major implications for cognitive functioning and overall well-being. Improving sleep duration and quality holds the potential to improve memory retention and cognitive performance. Individuals and healthcare professionals can use this information to create interventions and strategies that support healthy sleeping habits and improve memory outcomes. Despite the valuable insights provided by this systematic review, further research is required to investigate the association between REM sleep and memory retention. Conflicting findings in the literature necessitate more research on the impact of REM sleep on different types of memory. This comprehensive review emphasizes the importance of sleep patterns in memory consolidation. Individuals can improve their sleep patterns and boost their cognitive capacities by understanding and prioritizing the relevance of sleep for memory retention. More research in this area will lead to a greater understanding of the complex interaction between sleep and memory, ultimately leading to advancements in memory enhancement strategies and cognitive therapy.
